# Oscillations in deviating difference equations using an iterative technique

**DOI:** 10.1186/s13660-017-1450-8

**Published:** 2017-07-26

**Authors:** George E Chatzarakis, Irena Jadlovská

**Affiliations:** 1 Department of Electrical and Electronic Engineering Educators, School of Pedagogical and Technological Education (ASPETE), N. Heraklio, Athens, 14121 Greece; 2 Department of Mathematics and Theoretical Informatics, Faculty of Electrical Engineering and Informatics, Technical University of Košice, Letná 9, Košice, 042 00 Slovakia

**Keywords:** 39A10, 39A21, difference equations, non-monotone argument, retarded argument, advanced argument, oscillation, Grönwall inequality

## Abstract

The paper deals with the oscillation of the first-order linear difference equation with deviating argument and nonnegative coefficients. New sufficient oscillation conditions, involving limsup, are given, which essentially improve all known results, based on an iterative technique. We illustrate the results and the improvement over other known oscillation criteria by examples, numerically solved in Matlab.

## Introduction

Consider the difference equation with a variable retarded argument of the form

$$ \Delta x(n)+p(n)x\bigl(\tau(n)\bigr)=0, \quad n\in \mathbb{N} _{0},\hspace{192pt} (\mbox{E}) $$

and the (dual) difference equation with a variable advanced argument of the form

$$ \nabla x(n)-q(n)x\bigl(\sigma(n)\bigr)=0, \quad n\in \mathbb{N} , \hspace{192pt} \bigl(\mbox{E}^{\prime}\bigr) $$

where $\mathbb{N}_{0}$ and $\mathbb{N}$ are the sets of nonnegative integers and positive integers, respectively.

Equations (E) and ($\mathrm{E}^{\prime}$) are studied under the following assumptions: everywhere $(p(n))_{n\geq0}$ and $(q(n))_{n\geq1}$ are sequences of nonnegative real numbers, $(\tau(n))_{n\geq0}$ is a sequence of integers such that

1.1 $$ \tau(n)\leq n-1,\quad\forall n\in \mathbb{N} _{0}\quad \text{and}\quad\lim_{n\rightarrow\infty}\tau(n)=\infty $$

and $(\sigma(n))_{n\geq1}$ is a sequence of integers such that

1.2 $$ \sigma(n)\geq n+1,\quad\forall n\in \mathbb{N} . $$

Here, Δ denotes the forward difference operator $\Delta x(n)=x(n+1)-x(n)$ and ∇ corresponds to the backward difference operator $\nabla x(n)=x(n)-x(n-1)$.

Set $w=-\min_{n\geq0}\tau(n)$. Clearly, *w* is a finite positive integer if (1.1) holds.

By a *solution* of (E), we mean a sequence of real numbers $(x(n))_{n\geq-w}$ which satisfies (E) for all $n\geq 0$. It is clear that, for each choice of real numbers $c_{-w}$, $c_{-w+1}$, … , $c_{-1}$, $c_{0}$, there exists a unique solution $(x(n))_{n\geq-w}$ of (E) which satisfies the initial conditions $x(-w)=c_{-w}$, $x(-w+1)=c_{-w+1}$, … , $x(-1)=c_{-1}$, $x(0)=c_{0}$. When the initial data is given, we can obtain a unique solution to (E) by using the method of steps.

By a solution of ($\mathrm{E}^{\prime}$), we mean a sequence of real numbers $( x(n) ) _{n\geq0}$ which satisfies ($\mathrm{E}^{\prime}$) for all $n\geq1$.

A solution $(x(n))_{n\geq-w}$ (or $( x(n) ) _{n\geq0}$) of (E) (or ($\mathrm{E}^{\prime}$)) is called *oscillatory*, if the terms $x(n)$ of the sequence are neither eventually positive nor eventually negative. Otherwise, the solution is said to be *nonoscillatory*. An equation is *oscillatory* if all its solutions oscillate.

In the last few decades, the oscillatory behavior and the existence of positive solutions of difference equations with deviating arguments have been extensively studied; see, for example, papers [1–20] and the references cited therein. Most of these papers concern the special case where the arguments are nondecreasing, while a small number of these papers are dealing with the general case where the arguments are non-monotone. See, for example, [1–3, 7, 8, 16] and the references cited therein. The consideration of non-monotone arguments of other than pure mathematical interest can be justified by the fact that it approximates (in a more accurate way) the natural phenomena described by an equation of type (E) or ($\mathrm{E}^{\prime}$). That is because there are always natural disturbances (*e.g.* noise in communication systems) that affect all the parameters of the equation and therefore the fair (from a mathematical point of view) monotone arguments become non-monotone almost always. In view of this, for the case of equation (E) (or ($\mathrm{E}^{\prime }$)) an interesting question arising is whether we can state oscillation criteria considering the argument $\tau(n)$ (or $\sigma(n)$) to be not necessarily monotone. In the present paper, we achieve this goal by establishing criteria which, up to our knowledge, essentially improve all other known results in the literature.

Throughout this paper, we are going to use the following notations:

1.3 $$\begin{aligned}& \sum_{i=k}^{k-1}A(i)=0\quad\text{and}\quad \prod_{i=k}^{k-1}A(i)=1, \quad\text{where } A(i) \in\mathbb{R}_{+}, \\& \alpha=\liminf_{n\rightarrow\infty}\sum _{j=\tau(n)}^{n-1}p(j) , \end{aligned}$$

1.4 $$\begin{aligned}& \beta=\liminf_{n\rightarrow\infty}\sum _{j=n+1}^{\sigma(n)}q(j) , \\& D(\omega):= \textstyle\begin{cases} 0, & \text{if }\omega>1/e, \\ \frac{1-\omega-\sqrt{1-2\omega-\omega^{2}}}{2}, & \text{if }\omega \in [ 0,1/e ], \end{cases}\displaystyle \\& \mathit{MD}:=\limsup_{n\rightarrow\infty}\sum _{j=h(n)}^{n}p(j), \\& \mathit{MA}:=\limsup_{n\rightarrow\infty}\sum _{j=n}^{\rho(n)}q(j), \end{aligned}$$

where

1.5 $$ h(n)=\max_{0\leq s\leq n}\tau(s) $$

and

1.6 $$ \rho(n)=\min_{s\geq n}\sigma(s). $$

Clearly, the sequences $h(n)$ and $\rho(n)$ are nondecreasing with $\tau (n)\leq h(n)\leq n-1$ for all $n\geq0$ and $\sigma(n)\geq\rho (n)\geq n+1$ for all $n\geq1$, respectively.

### Chronological review for retarded difference equations

In 2008, Chatzarakis, Koplatadze and Stavroulakis [4, 5] proved that if

1.7 $$ \mathit{MD}>1 $$

or

1.8 $$ \alpha>\frac{1}{e}, $$

then all solutions of (E) oscillate.

It is obvious that there is a gap between the conditions (1.7) and (1.8) when the limit

$$ \lim_{n\rightarrow\infty}\sum_{j=\tau(n)}^{n-1}p(j) $$

does not exist. How to fill this gap is an interesting problem which has been investigated by several authors. For example, in 2009, Chatzarakis, Philos and Stavroulakis [6] proved that if

1.9 $$ \mathit{MD}>1-D(\alpha), $$

then all solutions of (E) oscillate.

In 2011, Braverman and Karpuz [3] proved that if

1.10 $$ \limsup_{n\rightarrow\infty }\sum_{j=h(n)}^{n}p(j) \prod_{i=\tau(j)}^{h(n)-1}\frac{1}{1-p(i)}>1, $$

then all solutions of (E) oscillate, while, in 2014, Stavroulakis [16] improved (1.10) to

1.11 $$ \limsup_{n\rightarrow\infty }\sum_{j=h(n)}^{n}p(j) \prod_{i=\tau(j)}^{h(n)-1}\frac {1}{1-p(i)}>1-D( \alpha). $$

In 2015, Braverman, Chatzarakis and Stavroulakis [2] proved that if for some $r\in{\mathbb{N}}$

1.12 $$ \limsup_{n\rightarrow\infty }\sum_{j=h(n)}^{n}p(j)a_{r}^{-1} \bigl( h(n),\tau(j) \bigr) >1, $$

or

1.13 $$ \limsup_{n\rightarrow\infty }\sum_{j=h(n)}^{n}p(j)a_{r}^{-1} \bigl( h(n),\tau(j) \bigr) >1-D(\alpha), $$

where

1.14 $$ a_{1}(n,k)=\prod_{i=k}^{n-1} \bigl[ 1-p(i) \bigr] ,\qquad a_{r+1}(n,k)=\prod _{i=k}^{n-1} \bigl[ 1-p(i)a_{r}^{-1} \bigl(i,\tau(i)\bigr) \bigr] , $$

then all solutions of (E) oscillate.

Recently, Asteris and Chatzarakis [1], and Chatzarakis and Shaikhet [8] proved that if for some $\ell\in \mathbb{N} $

1.15 $$ \limsup_{n\rightarrow\infty }\sum_{i=h(n)}^{n}p(i) \prod_{j=\tau(i)}^{h(n)-1}\frac{1}{1-p_{\ell}(j)}>1 $$

or

1.16 $$ \limsup_{n\rightarrow\infty }\sum_{i=h(n)}^{n}p(i) \prod_{j=\tau(i)}^{h(n)-1}\frac{1}{1-p_{\ell }(j)}>1-D( \alpha), $$

where

1.17 $$ p_{\ell}(n)=p(n) \Biggl[ 1+\sum _{i=\tau (n)}^{n-1}p(i)\prod_{j=\tau(i)}^{h(n)-1} \frac{1}{1-p_{\ell -1}(j)} \Biggr] $$

with $p_{0}(n)=p(n)$, then all solutions of (E) oscillate.

Lately, Chatzarakis, Pournaras and Stavroulakis [7] proved that if for some $\ell\in \mathbb{N} $

1.18 $$ \limsup_{n\rightarrow\infty }\sum_{i=h(n)}^{n}p(i) \prod_{j=\tau(i)}^{h(n)-1}\frac{1}{1-P_{\ell}(j)}>1, $$

or

1.19 $$ \limsup_{n\rightarrow\infty }\sum_{i=h(n)}^{n}p(i) \prod_{j=\tau(i)}^{h(n)-1}\frac{1}{1-P_{\ell }(j)}>1-D( \alpha), $$

or

1.20 $$ \limsup_{n\rightarrow\infty }\sum_{i=h(n)}^{n}p(i) \prod_{j=\tau(i)}^{n}\frac{1}{1-P_{\ell }(j)}> \frac{1}{D(\alpha)}, $$

where

1.21 $$ P_{\ell}(n)=p(n) \Biggl[ 1+\sum _{i=\tau(n)}^{n-1}p(i)\exp \Biggl( \sum _{j=\tau(i)}^{n-1}p(j)\prod_{m=\tau(j)}^{j-1} \frac{1}{1-P_{\ell -1}(m)} \Biggr) \Biggr] $$

with $P_{0}(n)=p(n)$, then all solutions of (E) are oscillatory.

### Chronological review for advanced difference equations

In 2012, Chatzarakis and Stavroulakis [9] proved that if

1.22 $$ \mathit{MA}>1, $$

or

1.23 $$ \mathit{MA}>1- ( 1-\sqrt{1-\beta} ) ^{2}, $$

then all solutions of ($\mathrm{E}^{\prime}$) oscillate.

In 2015, Braverman, Chatzarakis and Stavroulakis [2] proved that if for some $r\in{\mathbb{N}}$

1.24 $$ \limsup_{n\rightarrow\infty}\sum_{j=n}^{\rho (n)}q(j)b_{r}^{-1} \bigl(\rho(n),\sigma(j)\bigr)>1, $$

or

1.25 $$ \limsup_{n\rightarrow\infty}\sum_{j=n}^{\rho (n)}q(j)b_{r}^{-1} \bigl(\rho(n),\sigma(j)\bigr)>1-D(\beta), $$

where

1.26 $$ b_{1}(n,k)=\prod_{i=n+1}^{k} \bigl[ 1-q(i) \bigr] , \qquad b_{r+1}(n,k)=\prod _{i=n+1}^{k} \bigl[ 1-q(i)b_{r}^{-1} \bigl(i,\sigma (i)\bigr) \bigr] $$

then all solutions of ($\mathrm{E}^{\prime}$) oscillate.

Recently, Asteris and Chatzarakis [1], and Chatzarakis and Shaikhet [8] proved that if for some $\ell\in \mathbb{N} $

1.27 $$ \limsup_{n\rightarrow\infty}\sum_{i=n}^{\rho (n)}q(i) \prod_{j=\rho(n)+1}^{\sigma(i)}\frac{1}{1-q_{\ell}(j)}>1, $$

or

1.28 $$ \limsup_{n\rightarrow\infty}\sum_{i=n}^{\rho (n)}q(i) \prod_{j=\rho(n)+1}^{\sigma(i)}\frac{1}{1-q_{\ell }(j)}>1-D( \beta), $$

where

1.29 $$ q_{\ell}(n)=q(n) \Biggl[ 1+\sum _{i=n+1}^{\rho (n)}q(i)\prod_{j=\rho(n)+1}^{\sigma(i)} \frac{1}{1-q_{\ell -1}(j)} \Biggr] $$

with $q_{0}(n)=q(n)$, then all solutions of ($\mathrm{E}^{\prime}$) oscillate.

## Main results and discussion

### Main results

We study further (E) and ($\mathrm{E}^{\prime}$), and derive new sufficient oscillation conditions, involving limsup, which essentially improve all the previous results.

#### Retarded difference equations

The following simple result is stated to explain why we can consider only the case

2.1 $$ p(n)< \frac{1}{\lambda_{0}}, \quad\forall n\geq0 , $$

where $\lambda_{0}>1$ is the smaller root of the transcendental equation $\lambda=e^{\alpha\lambda}$ with $0<\alpha\leq1/e$.

##### Theorem 1

*Assume that there exists a subsequence* $\theta(n)$, $n\in \mathbb{N} $ *of positive integers such that*

2.2 $$ p\bigl(\theta(n)\bigr)\geq\frac{1}{\lambda_{0}}, \quad\forall n\in{ \mathbb{N}}. $$

*Then all solutions of* (E) *are oscillatory*.

##### Proof

Assume, for the sake of contradiction, that $( x(n) ) _{n\geq-w}$ is a nonoscillatory solution of (E). Then it is either eventually positive or eventually negative. As $( -x(n) ) _{n\geq-w}$ is also a solution of (E), we may restrict ourselves only to the case where $x(n)>0$ for all large *n*. Let $n_{1}\geq-w$ be an integer such that $x(n)>0$ for all $n\geq n_{1}$. Then there exists $n_{2}\geq n_{1}$ such that $x(\tau(n))>0$, $\forall n\geq n_{2}$. In view of this, equation (E) becomes

$$ \Delta x(n)=-p(n)x\bigl(\tau(n)\bigr)\leq0, \quad\forall\theta(n)\geq n_{2}, $$

which means that the sequence $(x(n))$ is eventually nonincreasing.

Taking into account the fact that (2.2) holds, equation (E) gives

$$\begin{aligned} x\bigl(\theta(n)+1\bigr) =&x\bigl(\theta(n)\bigr)-p\bigl(\theta(n)\bigr)x\bigl( \tau\bigl(\theta(n)\bigr)\bigr) \\ \leq&\frac{1}{\lambda_{0}}x\bigl(\theta(n)\bigr)-p\bigl(\theta(n)\bigr)x\bigl(\tau \bigl(\theta(n)\bigr)\bigr) \\ \leq&\frac{1}{\lambda_{0}}x\bigl(\theta(n)\bigr)-x\bigl(\theta(n)\bigr)p\bigl( \theta(n)\bigr) \\ =&x\bigl(\theta(n)\bigr) \biggl( \frac{1}{\lambda_{0}}-p\bigl(\theta(n)\bigr) \biggr) \leq 0, \quad \text{for all } \theta(n)\geq n_{2}, \end{aligned}$$

where $\theta(n)\rightarrow\infty$ as $n\rightarrow\infty$, which contradicts the assumption that $x(n)>0$ for all $n\geq n_{1}$. □

The proofs of our main results are essentially based on the following lemmas.

The first lemma is taken from [8]. For the sake of completeness, we cite its proof here.

##### Lemma 1

[8], Lemma 1

*Assume that* (1.1) *holds and* *α* *is defined by* (1.3) *with* $\alpha>0$. *Then*

2.3 $$ \liminf_{n\rightarrow\infty }\sum_{j=h(n)}^{n-1}p(j)= \liminf_{n\rightarrow\infty }\sum_{j=\tau(n)}^{n-1}p(j)= \alpha, $$

*where* $h(n)$ *is defined by* (1.5).

##### Proof

Since $h(n)$ is nondecreasing and $\tau(n)\leq h(n)\leq n-1$ for all $n\geq 0$, we have

$$ \sum_{j=h(n)}^{n-1}p(j)\leq\sum _{j=\tau(n)}^{n-1}p(j). $$

Therefore

$$ \liminf_{n\rightarrow\infty}\sum_{j=h(n)}^{n-1}p(j) \leq \liminf_{n\rightarrow\infty}\sum_{j=\tau(n)}^{n-1}p(j). $$

If (2.3) does not hold, then there exist $\alpha^{\prime}>0$ and a subsequence $( \theta(n) ) $ such that $\theta(n)\rightarrow \infty$ as $n\rightarrow\infty$ and

$$ \lim_{n\rightarrow\infty}\sum_{j=h(\theta(n))}^{\theta(n)-1}p(j) \leq \alpha^{\prime}< \alpha. $$

But $h(\theta(n))=\max_{0\leq s\leq\theta(n)}\tau(s)$, hence there exists $\theta^{\prime}(n)\leq\theta(n)$, $\theta^{\prime}(n)\in \mathbb{N}_{0}$, such that $h(\theta(n))=\tau(\theta^{\prime}(n))$, and consequently

$$ \sum_{j=h(\theta(n))}^{\theta(n)-1}p(j)=\sum _{j=\tau(\theta^{\prime }(n))}^{\theta(n)-1}p(j)\geq\sum_{j=\tau(\theta^{\prime }(n))}^{\theta ^{\prime}(n)-1}p(j). $$

It follows that $( \sum_{j=\tau(\theta^{\prime}(n))}^{\theta ^{\prime}(n)-1}p(j) ) _{n=1}^{\infty}$ is a bounded sequence having a convergent subsequence, say

$$ \sum_{j=\tau(\theta^{\prime}(n_{k}))}^{\theta^{\prime }(n_{k})-1}p(j)\rightarrow c\leq \alpha^{\prime},\quad \text{as }k\rightarrow\infty, $$

which implies that

$$ \liminf_{n\rightarrow\infty}\sum_{j=\tau(n)}^{n-1}p(j) \leq\alpha ^{\prime}< \alpha. $$

This contradicts (2.3).

The proof of the lemma is complete. □

##### Lemma 2

[6], Lemma 2.1

*Assume that* (1.1) *holds*, $h(n)$ *is defined by* (1.5), $0<\alpha\leq1/e$ *and* $x(n)$ *is an eventually positive solution of* (E). *Then*

2.4 $$ \liminf_{n\rightarrow\infty}\frac{x(n+1)}{x(h(n))}\geq D(\alpha). $$

##### Lemma 3

*Assume that* (1.1) *holds*, $h(n)$ *is defined by* (1.5), $0<\alpha\leq1/e$ *and* $x(n)$ *is an eventually positive solution of* (E). *Then*

2.5 $$ \liminf_{n\rightarrow\infty}\frac{x(h(n))}{x(n)}\geq \lambda_{0}, $$

*where* $\lambda_{0}$ *is the smaller root of the transcendental equation* $\lambda=e^{\alpha\lambda}$.

##### Proof

Assume that $(x(n))_{n\geq-w}$ is an eventually positive solution of (E). Then there exists $n_{1}\geq-w$ such that $x(n)$, $x(\tau (n))>0$ for all $n\geq n_{1}$. In view of this, equation (E) becomes

$$ \Delta x(n)=-p(n)x\bigl(\tau(n)\bigr)\leq0, \quad\forall n\geq n_{1}, $$

which means that $(x(n))$ is an eventually nonincreasing sequence of positive numbers.

Taking into account that $0<\alpha\leq1/e$, it is clear that there exists $\varepsilon\in ( 0,\alpha ) $ such that

$$ \sum_{j=h(n)}^{n-1}p(j)\geq\alpha-\varepsilon \quad \text{for }n\geq n ( \varepsilon ) \geq n_{1}. $$

We will show that

2.6 $$ \liminf_{n\rightarrow\infty}\frac{x(h(n))}{x(n)}\geq \lambda_{0} ( \varepsilon ) , $$

where $\lambda_{0} ( \varepsilon ) $ is the smaller root of the equation

$$ e^{ ( \alpha-\varepsilon ) \lambda}=\lambda. $$

Assume, for the sake of contradiction, that (2.6) is incorrect. Then there exists $\varepsilon_{0}>0$ such that

2.7 $$ \frac{e^{ ( \alpha-\varepsilon ) \gamma}}{\gamma}\geq 1+\varepsilon_{0}, $$

where

2.8 $$ \gamma=\liminf_{n\rightarrow\infty}\frac{x(h(n))}{x(n)}< \lambda _{0} ( \varepsilon ) . $$

On the other hand, for any $\delta>0$ there exists $n ( \delta ) $ such that

2.9 $$ \frac{x(h(n))}{x(n)}\geq\gamma-\delta\quad \text{for }n\geq n ( \delta ) . $$

Dividing (E) by $x(n)$ we obtain

$$\begin{aligned} \frac{\Delta x(n)}{x(n)} =&-p(n)\frac{x(\tau(n))}{x(n)} \\ \leq&-\frac{x(h(n))}{x(n)}p(n)\leq- ( \gamma-\delta ) p(n), \end{aligned}$$

or

$$ \frac{\Delta x(n)}{x(n)}\leq- ( \gamma-\delta ) p(n). $$

Summing up last inequality from $h(n)$ to $n-1$, we get

2.10 $$ \sum_{j=h(n)}^{n-1} \frac{\Delta x(j)}{x(j)}\leq- ( \gamma-\delta ) \sum_{j=h(n)}^{n-1}p(j) \leq- ( \gamma-\delta ) ( \alpha-\varepsilon ) . $$

But, since $e^{x}\geq x+1$, $\forall x>0$ we have

$$\begin{aligned} \sum_{j=h(n)}^{n-1}\frac{\Delta x(j)}{x(j)} =&\sum _{j=h(n)}^{n-1} \biggl( \frac{x(j+1)}{x(j)}-1 \biggr) \\ =&\sum_{j=h(n)}^{n-1}\exp \biggl( \ln \frac{x(j+1)}{x(j)} \biggr) - \bigl( n-h(n) \bigr) \\ \geq&\sum_{j=h(n)}^{n-1} \biggl( 1+\ln \frac{x(j+1)}{x(j)} \biggr) - \bigl( n-h(n) \bigr) \\ =& \bigl( n-h(n) \bigr) +\sum_{j=h(n)}^{n-1}\ln \frac {x(j+1)}{x(j)}- \bigl( n-h(n) \bigr) \\ =&\sum_{j=h(n)}^{n-1}\ln\frac{x(j+1)}{x(j)}=\ln \frac{x(n)}{x(h(n))}, \end{aligned}$$

or

2.11 $$ \sum_{j=h(n)}^{n-1} \frac{\Delta x(j)}{x(j)}\geq\ln\frac{x(n)}{x(h(n))}. $$

Combining (2.10) and (2.11), we have

$$ \ln\frac{x(n)}{x(h(n))}\leq- ( \gamma-\delta ) ( \alpha -\varepsilon ) , $$

*i.e.*,

$$ \frac{x(h(n))}{x(n)}\geq e^{ ( \gamma-\delta ) ( \alpha -\varepsilon ) }\quad\text{for }n\geq n ( \delta ) . $$

Therefore,

$$ \gamma=\liminf_{t\rightarrow\infty}\frac{x(h(n))}{x(n)}\geq e^{ ( \gamma-\delta ) ( \alpha-\varepsilon ) }, $$

which, as $\delta\rightarrow0$, implies

$$ \gamma\geq e^{\gamma ( \alpha-\varepsilon ) }. $$

Combining the last inequality with (2.7), we obtain

$$ \frac{e^{\gamma ( \alpha-\varepsilon ) }}{1+\varepsilon _{0}}\geq e^{\gamma ( \alpha-\varepsilon ) }, $$

which is impossible since $\varepsilon_{0}>0$. Therefore (2.6) is true. Since $\lambda_{0} ( \varepsilon ) \rightarrow\lambda_{0}$ as $\varepsilon\rightarrow0$, (2.6) implies (2.5).

The proof of the lemma is complete. □

##### Theorem 2

*Assume that* (1.1) *and* (2.1) *hold*, *and* $h(n)$ *is defined by* (1.5). *If for some* $\ell\in \mathbb{N} $

2.12 $$ \limsup_{n\rightarrow\infty}\sum_{i=h(n)}^{n}p(i) \exp \Biggl( \sum_{j=\tau(i)}^{h(n)-1}p(j)\prod _{m=\tau(j)}^{j-1}\frac {1}{1-\widetilde{P}_{\ell}(m)} \Biggr) >1, $$

*where*

2.13 $$ \widetilde{P}_{\ell}(n)=p(n) \Biggl[ 1+\sum _{i=\tau (n)}^{n-1}p(i)\exp \Biggl( \sum _{j=\tau (i)}^{n-1}p(j)\prod_{m=\tau(j)}^{j-1} \frac{1}{1-\widetilde{P}_{\ell -1}(m)} \Biggr) \Biggr] $$

*with* $\widetilde{P}_{0}(n)=\lambda_{0}p(n)$ *and* $\lambda_{0}$ *is the smaller root of the transcendental equation* $\lambda=e^{\alpha\lambda}$, *then all solutions of* (E) *are oscillatory*.

##### Proof

Assume that $(x(n))_{n\geq-w}$ is an eventually positive solution of (E). Then there exists $n_{1}\geq-w$ such that $x(n)$, $x(\tau (n))>0$ for all $n\geq n_{1}$. In view of this, equation (E) becomes

$$ \Delta x(n)=-p(n)x\bigl(\tau(n)\bigr)\leq0,\quad\forall n\geq n_{1}, $$

which means that $(x(n))$ is an eventually nonincreasing sequence of positive numbers.

Taking this into account along with the fact that $\tau(n)\leq h(n)$, (E) implies

2.14 $$ \Delta x(n)+p(n)x\bigl(h(n)\bigr)\leq0, \quad n\geq n_{1}. $$

Observe that (2.5) implies that for each $\epsilon>0$ there exists a $n(\epsilon)$ such that

2.15 $$ \frac{x(h(n))}{x(n)}>\lambda_{0}-\epsilon, \quad \text{for all }n\geq n(\epsilon)\geq n_{1}. $$

Combining the inequalities (2.14) and (2.15) we obtain

$$ \Delta x(n)+p(n) ( \lambda_{0}-\epsilon ) x(n)< 0, \quad n\geq n( \epsilon), $$

or

2.16 $$ \Delta x(n)+\widetilde{P}_{0}(n,\epsilon)x(n)< 0, \quad n\geq n(\epsilon), $$

where

$$ \widetilde{P}_{0}(n,\epsilon)= ( \lambda_{0}-\epsilon ) p(n). $$

Applying the discrete Grönwall inequality, we obtain

2.17 $$ x(k)>x(n)\prod_{i=k}^{n-1} \frac{1}{1-\widetilde{P}_{0}(i,\epsilon )},\quad\text{for all }n\geq n(\epsilon). $$

Dividing (E) by $x(n)$ and summing up from *k* to $n-1$, we take

2.18 $$ \sum_{j=k}^{n-1} \frac{\Delta x(j)}{x(j)}=-\sum_{j=k}^{n-1}p(j) \frac {x(\tau(j))}{x(j)}. $$

Also, since $e^{x}\geq x+1$, $x>0$ we have

$$\begin{aligned} \sum_{j=k}^{n-1}\frac{\Delta x(j)}{x(j)} =&\sum _{j=k}^{n-1} \biggl( \frac {x(j+1)}{x(j)}-1 \biggr) \\ =&\sum_{j=k}^{n-1} \biggl[ \exp \biggl( \ln \frac{x(j+1)}{x(j)} \biggr) -1 \biggr] \\ \geq&\sum_{j=k}^{n-1} \biggl[ \ln \frac{x(j+1)}{x(j)}+1-1 \biggr] \\ =&\sum_{j=k}^{n-1}\ln\frac{x(j+1)}{x(j)}=\ln \frac{x(n)}{x(k)}, \end{aligned}$$

or

2.19 $$ \sum_{j=k}^{n-1} \frac{\Delta x(j)}{x(j)}\geq\ln\frac{x(n)}{x(k)}. $$

Combining (2.18) and (2.19), we obtain

$$ -\sum_{j=k}^{n-1}p(j)\frac{x(\tau(j))}{x(j)}\geq \ln\frac{x(n)}{x(k)}, $$

or

2.20 $$ \ln\frac{x(k)}{x(n)}\geq\sum_{j=k}^{n-1}p(j) \frac{x(\tau(j))}{x(j)}. $$

Since $\tau(j)< j$, (2.17) implies

2.21 $$ x\bigl(\tau(j)\bigr)>x(j)\prod_{i=\tau(j)}^{j-1} \frac{1}{1-\widetilde {P}_{0}(i,\epsilon)}. $$

In view of (2.21), (2.20) gives

$$ \ln\frac{x(k)}{x(n)}>\sum_{j=k}^{n-1}p(j) \prod_{i=\tau (j)}^{j-1}\frac{1}{1-\widetilde{P}_{0}(i,\epsilon)}, $$

or

2.22 $$ x(k)>x(n)\exp \Biggl( \sum_{j=k}^{n-1}p(j) \prod_{i=\tau (j)}^{j-1}\frac{1}{1-\widetilde{P}_{0}(i,\epsilon)} \Biggr) . $$

Summing up (E) from $\tau(n)$ to $n-1$, we have

2.23 $$ x(n)-x\bigl(\tau(n)\bigr)+\sum_{i=\tau(n)}^{n-1}p(i)x \bigl( \tau(i) \bigr) =0. $$

Setting $k=\tau ( i ) $ in (2.22) implies

2.24 $$ x\bigl(\tau(i)\bigr)>x(n)\exp \Biggl( \sum _{j=\tau (i)}^{n-1}p(j)\prod_{m=\tau(j)}^{j-1} \frac{1}{1-\widetilde {P}_{0}(m,\epsilon)} \Biggr) , $$

so, combining (2.23) and (2.24), we find

$$ x(n)-x\bigl(\tau(n)\bigr)+x(n)\sum_{i=\tau(n)}^{n-1}p(i) \exp \Biggl( \sum_{j=\tau(i)}^{n-1}p(j)\prod _{m=\tau(j)}^{j-1}\frac{1}{1-\widetilde {P}_{0}(m,\epsilon)} \Biggr) < 0. $$

Multiplying the last inequality by $p(n)$, we get

$$ p(n)x(n)-p(n)x\bigl(\tau(n)\bigr) +p(n)x(n)\sum_{i=\tau(n)}^{n-1}p(i) \exp \Biggl( \sum_{j=\tau(i)}^{n-1}p(j)\prod _{m=\tau(j)}^{j-1}\frac {1}{1-\widetilde{P}_{0}(m,\epsilon)} \Biggr) < 0, $$

which, in view of (E), becomes

$$ \Delta x(n)+p(n)x(n) +p(n)x(n)\sum_{i=\tau(n)}^{n-1}p(i) \exp \Biggl( \sum_{j=\tau(i)}^{n-1}p(j)\prod _{m=\tau(j)}^{j-1}\frac {1}{1-\widetilde{P}_{0}(m,\epsilon)} \Biggr) < 0, $$

*i.e.*,

$$ \Delta x(n)+p(n) \Biggl[ 1+\sum_{i=\tau(n)}^{n-1}p(i) \exp \Biggl( \sum_{j=\tau(i)}^{n-1}p(j)\prod _{m=\tau(j)}^{j-1}\frac{1}{1-\widetilde {P}_{0}(m,\epsilon)} \Biggr) \Biggr] x(n)< 0. $$

Therefore

2.25 $$ \Delta x(n)+\widetilde{P}_{1}(n,\epsilon)x(n)< 0, $$

where

$$ \widetilde{P}_{1}(n,\epsilon)=p(n) \Biggl[ 1+\sum _{i=\tau (n)}^{n-1}p(i)\exp \Biggl( \sum _{j=\tau (i)}^{n-1}p(j)\prod_{m=\tau(j)}^{j-1} \frac{1}{1-\widetilde {P}_{0}(m,\epsilon)} \Biggr) \Biggr] . $$

Repeating the above argument leads to a new estimate,

$$ \Delta x(n)+\widetilde{P}_{2}(n,\epsilon)x(n)< 0, $$

where

$$ \widetilde{P}_{2}(n,\epsilon)=p(n) \Biggl[ 1+\sum _{i=\tau (n)}^{n-1}p(i)\exp \Biggl( \sum _{j=\tau (i)}^{n-1}p(j)\prod_{m=\tau(j)}^{j-1} \frac{1}{1-\widetilde {P}_{1}(m,\epsilon)} \Biggr) \Biggr] . $$

Continuing by induction, for sufficiently large *n* we get

2.26 $$ \Delta x(n)+\widetilde{P}_{\ell}(n,\epsilon)x(n)< 0, $$

where

$$ \widetilde{P}_{\ell}(n,\epsilon)=p(n) \Biggl[ 1+\sum _{i=\tau (n)}^{n-1}p(i)\exp \Biggl( \sum _{j=\tau (i)}^{n-1}p(j)\prod_{m=\tau(j)}^{j-1} \frac{1}{1-\widetilde{P}_{\ell -1}(m,\epsilon)} \Biggr) \Biggr] $$

and

2.27 $$ x\bigl(\tau(i)\bigr)>x\bigl(h(n)\bigr)\exp \Biggl( \sum _{j=\tau (i)}^{h(n)-1}p(j)\prod_{m=\tau(j)}^{j-1} \frac{1}{1-\widetilde{P}_{\ell }(m,\epsilon)} \Biggr) . $$

Summing up (E) from $h(n)$ to *n*, we have

2.28 $$ x(n+1)-x\bigl(h(n)\bigr)+\sum_{i=h(n)}^{n}p(i)x \bigl(\tau(i)\bigr)=0. $$

Combining (2.27) and (2.28), we have, for all sufficiently large *n*,

2.29 $$ \begin{aligned}[b] &x(n+1)-x\bigl(h(n)\bigr) \\ &\quad {}+x\bigl(h(n)\bigr)\sum_{i=h(n)}^{n}p(i) \exp \Biggl( \sum_{j=\tau (i)}^{h(n)-1}p(j)\prod _{m=\tau(j)}^{j-1}\frac{1}{1-\widetilde{P}_{\ell }(m,\epsilon)} \Biggr) < 0. \end{aligned} $$

The inequality is valid if we omit $x(n+1)>0$ in the left-hand side:

$$ -x\bigl(h(n)\bigr)+x\bigl(h(n)\bigr)\sum_{i=h(n)}^{n}p(i) \exp \Biggl( \sum_{j=\tau(i)}^{h(n)-1}p(j)\prod _{m=\tau(j)}^{j-1}\frac {1}{1-\widetilde{P}_{\ell}(m,\epsilon)} \Biggr) < 0. $$

Thus, as $x(h(n))>0$, for all sufficiently large *n*,

$$ \sum_{i=h(n)}^{n}p(i)\exp \Biggl( \sum _{j=\tau (i)}^{h(n)-1}p(j)\prod _{m=\tau(j)}^{j-1}\frac{1}{1-\widetilde{P}_{\ell }(m,\epsilon)} \Biggr) < 1, $$

from which by letting $n\rightarrow\infty$, we have

$$ \limsup_{n\rightarrow\infty}\sum_{i=h(n)}^{n}p(i) \exp \Biggl( \sum_{j=\tau(i)}^{h(n)-1}p(j)\prod _{m=\tau(j)}^{j-1}\frac {1}{1-\widetilde{P}_{\ell}(m,\epsilon)} \Biggr) \leq1. $$

Since *ϵ* may be taken arbitrarily small, this inequality contradicts (2.12).

The proof of the theorem is complete. □

##### Theorem 3

*Assume that* (1.1) *and* (2.1) *hold*, $h(n)$ *is defined by* (1.5) *and* $0<\alpha\leq1/e$. *If for some* $\ell\in \mathbb{N}$

2.30 $$ \limsup_{n\rightarrow\infty}\sum_{i=h(n)}^{n}p(i) \exp \Biggl( \sum_{j=\tau(i)}^{h(n)-1}p(j)\prod _{m=\tau(j)}^{j-1}\frac {1}{1-\widetilde{P}_{\ell}(m)} \Biggr) >1-D( \alpha), $$

*where* $\widetilde{P}_{\ell}(n)$ *is defined by* (2.13), *then all solutions of* (E) *are oscillatory*.

##### Proof

Assume, for the sake of contradiction, that $(x(n))_{n\geq-w}$ is an eventually positive solution of (E). Then, as in the proof of Theorem 1, for sufficiently large *n*, (2.29) is satisfied, *i.e.*,

$$ x(n+1)-x\bigl(h(n)\bigr) +x\bigl(h(n)\bigr)\sum_{i=h(n)}^{n}p(i) \exp \Biggl( \sum_{j=\tau(i)}^{h(n)-1}p(j)\prod _{m=\tau(j)}^{j-1}\frac {1}{1-\widetilde{P}_{\ell}(m,\epsilon)} \Biggr) < 0. $$

That is,

$$ \sum_{i=h(n)}^{n}p(i)\exp \Biggl( \sum _{j=\tau (i)}^{h(n)-1}p(j)\prod _{m=\tau(j)}^{j-1}\frac{1}{1-\widetilde{P}_{\ell }(m,\epsilon)} \Biggr) < 1- \frac{x(n+1)}{x(h(n))}, $$

which gives

$$ \limsup_{n\rightarrow\infty}\sum_{i=h(n)}^{n}p(i) \exp \Biggl( \sum_{j=\tau(i)}^{h(n)-1}p(j)\prod _{m=\tau(j)}^{j-1}\frac {1}{1-\widetilde{P}_{\ell}(m,\epsilon)} \Biggr) \leq 1- \liminf_{n\rightarrow\infty}\frac{x(n+1)}{x(h(n))}. $$

By Lemma 2, inequality (2.4) holds. So the last inequality leads to

$$ \limsup_{n\rightarrow\infty}\sum_{i=h(n)}^{n}p(i) \exp \Biggl( \sum_{j=\tau(i)}^{h(n)-1}p(j)\prod _{m=\tau(j)}^{j-1}\frac {1}{1-\widetilde{P}_{\ell}(m,\epsilon)} \Biggr) \leq1-D(\alpha). $$

Since *ϵ* may be taken arbitrarily small, this inequality contradicts (2.30).

The proof of the theorem is complete. □

##### Remark 1

It is clear that the left-hand sides of both conditions (2.12) and (2.30) are identical, also the right-hand side of condition (2.30) reduces to (2.12) in the case that $\alpha=0$. So it seems that Theorem 3 is the same as Theorem 2 when $\alpha=0$. However, one may notice that condition $0<\alpha\leq1/e$ is required in Theorem 3 but not in Theorem 2.

##### Theorem 4

*Assume that* (1.1) *and* (2.1) *hold*, $h(n)$ *is defined by* (1.5) *and* $0<\alpha\leq1/e$. *If for some* $\ell\in \mathbb{N}$

2.31 $$ \limsup_{n\rightarrow\infty}\sum_{i=h(n)}^{n}p(i) \exp \Biggl( \sum_{j=\tau(i)}^{n}p(j)\prod _{m=\tau(j)}^{j-1}\frac{1}{1-\widetilde {P}_{\ell}(m)} \Biggr) > \frac{1}{D(\alpha)}-1, $$

*where* $\widetilde{P}_{\ell}(n)$ *is defined by* (2.13), *then all solutions of* (E) *are oscillatory*.

##### Proof

Assume, for the sake of contradiction, that $(x(n))_{n\geq-w}$ is an eventually solution of (E). Then, as in the proof of Theorem 1, for sufficiently large *n*, (2.27) is satisfied. Therefore

2.32 $$ x\bigl(\tau(i)\bigr)>x(n+1)\exp \Biggl( \sum _{j=\tau (i)}^{n}p(j)\prod_{m=\tau(j)}^{j-1} \frac{1}{1-\widetilde{P}_{\ell }(m,\epsilon)} \Biggr) . $$

Summing up (E) from $h(n)$ to *n*, we have

$$ x(n+1)-x\bigl(h(n)\bigr)+\sum_{i=h(n)}^{n}p(i)x \bigl(\tau(i)\bigr)=0, $$

which, in view of (2.32), gives

$$ x(n+1)-x\bigl(h(n)\bigr) +\sum_{i=h(n)}^{n}p(i)x(n+1) \exp \Biggl( \sum_{j=\tau(i)}^{n}p(j)\prod _{m=\tau(j)}^{j-1}\frac{1}{1-\widetilde {P}_{\ell}(m,\epsilon)} \Biggr) < 0, $$

or

$$ x(n+1)-x\bigl(h(n)\bigr) +x\bigl(h(n)\bigr)\sum_{i=h(n)}^{n}p(i) \frac{x(n+1)}{x(h(n))}\exp \Biggl( \sum_{j=\tau(i)}^{n}p(j) \prod_{m=\tau (j)}^{j-1}\frac{1}{1-\widetilde{P}_{\ell}(m,\epsilon)} \Biggr) < 0. $$

Thus, for all sufficiently large *n*,

$$ \sum_{i=h(n)}^{n}p(i)\exp \Biggl( \sum _{j=\tau (i)}^{n}p(j)\prod _{m=\tau(j)}^{j-1}\frac{1}{1-\widetilde{P}_{\ell }(m,\epsilon)} \Biggr) < \frac{x(h(n))}{x(n+1)}-1. $$

Letting $n\rightarrow\infty$, we take

$$ \limsup_{n\rightarrow\infty}\sum_{i=h(n)}^{n}p(i) \exp \Biggl( \sum_{j=\tau(i)}^{n}p(j)\prod _{m=\tau(j)}^{j-1}\frac{1}{1-\widetilde {P}_{\ell}(m,\epsilon)} \Biggr) \leq\limsup _{n\rightarrow \infty}\frac{x(h(n))}{x(n+1)}-1, $$

which, in view of (2.4), gives

$$ \limsup_{n\rightarrow\infty}\sum_{i=h(n)}^{n}p(i) \exp \Biggl( \sum_{j=\tau(i)}^{n}p(j)\prod _{m=\tau(j)}^{j-1}\frac{1}{1-\widetilde {P}_{\ell}(m,\epsilon)} \Biggr) \leq \frac{1}{D(\alpha)}-1. $$

Since *ϵ* may be taken arbitrarily small, this inequality contradicts (2.31).

The proof of the theorem is complete. □

##### Remark 2

If $\widetilde{P}_{\ell}(n)\geq1$ then (2.26) guarantees that all solutions of (E) are oscillatory. In fact, (2.26) gives

$$ \Delta x(n)+x(n)\leq0, $$

which means that $x(n+1)\leq0$. This contradicts $x(n)>0$ for all $n\geq n_{1}$. Thus, in Theorems 2, 3 and 4 we consider only the case $\widetilde{P}_{\ell}(n)<1$. Another conclusion that can be drawn from the above, is that if at some point through the iterative process, we get a value of *ℓ*, for which $\widetilde{P}_{\ell}(n)\geq1$, then the process terminates, since in any case, all solutions of (E) will be oscillatory. The value of *ℓ*, that is, the number of iterations, obviously depends on the coefficient $p(n)$ and the form of the non-monotone argument $\tau(n)$.

#### Advanced difference equations

Similar oscillation theorems for the (dual) advanced difference equation ($\mathrm{E}^{\prime}$) can be derived easily. The proofs of these theorems are omitted, since they are quite similar to the proofs for a retarded equation.

The following simple result is stated to explain why we can consider only the case

2.33 $$ q(n)< \frac{1}{\lambda_{0}},\quad\forall n\in{\mathbb{N}}, $$

where $\lambda_{0}>1$ is the smaller root of the transcendental equation $\lambda=e^{\beta\lambda}$ with $0<\beta\leq1/e$.

##### Theorem 5

*Assume that there exists a subsequence* $\theta(n)$, $n\in \mathbb{N}$ *of positive integers such that*

2.34 $$ q\bigl(\theta(n)\bigr)\geq\frac{1}{\lambda_{0}},\quad\forall n\in{ \mathbb{N}}. $$

*Then all solutions of* ($\mathrm{E}^{\prime}$) *are oscillatory*.

##### Theorem 6

*Assume that* (1.2) *and* (2.33) *hold*, *and* $\rho (n)$ *is defined by* (1.6). *If for some* $\ell\in \mathbb{N} $

2.35 $$ \limsup_{n\rightarrow\infty}\sum_{i=n}^{\rho(n)}q(i) \exp \Biggl( \sum_{j=\rho(n)+1}^{\sigma(i)}q(j)\prod _{m=j+1}^{\sigma (j)}\frac{1}{1-\widetilde{Q}_{\ell}(m)} \Biggr) >1, $$

*where*

2.36 $$ \widetilde{Q}_{\ell}(n)=q(n) \Biggl[ 1+\sum _{i=n+1}^{\sigma (n)}q(i)\exp \Biggl( \sum _{j=n+1}^{\sigma (i)}q(j)\prod_{m=j+1}^{\sigma(j)} \frac{1}{1-\widetilde{Q}_{\ell -1}(m)} \Biggr) \Biggr] $$

*with* $\widetilde{Q}_{0}(n)=\lambda_{0}q(n)$ *and* $\lambda_{0}$ *is the smaller root of the transcendental equation* $\lambda=e^{\beta\lambda}$, *then all solutions of* ($\mathrm{E}^{\prime}$) *are oscillatory*.

##### Theorem 7

*Assume that* (1.2) *and* (2.33) *hold*, $\rho(n)$ *is defined by* (1.6) *and* $0<\beta\leq1/e$. *If for some* $\ell\in \mathbb{N} $

2.37 $$ \limsup_{n\rightarrow\infty}\sum_{i=n}^{\rho(n)}q(i) \exp \Biggl( \sum_{j=\rho(n)+1}^{\sigma(i)}q(j)\prod _{m=j+1}^{\sigma (j)}\frac{1}{1-\widetilde{Q}_{\ell}(m)} \Biggr) >1-D ( \beta ) , $$

*where* $\widetilde{Q}(n)$ *is defined by* (2.36), *then all solutions of* ($\mathrm{E}^{\prime}$) *are oscillatory*.

##### Remark 3

It is clear that the left-hand sides of both conditions (2.35) and (2.37) are identical, also the right hand side of condition (2.37) reduces to (2.35) in the case that $\beta =0$. So it seems that Theorem 7 is the same as Theorem 6 when $\beta =0 $. However, one may notice that condition $0<\beta\leq1/e$ is required in Theorem 7 but not in Theorem 6.

##### Theorem 8

*Assume that* (1.2) *and* (2.33) *hold*, $\rho(n)$ *is defined by* (1.6) *and* $0<\beta\leq1/e$. *If for some* $\ell\in \mathbb{N} $

2.38 $$ \limsup_{n\rightarrow\infty}\sum_{i=n}^{\rho(n)}q(i) \exp \Biggl( \sum_{j=n}^{\sigma(i)}q(j)\prod _{m=j+1}^{\sigma(j)}\frac {1}{1-\widetilde{Q}_{\ell}(m)} \Biggr) > \frac{1}{D(\beta)}-1, $$

*where* $\widetilde{Q}(n)$ *is defined by* (2.36), *then all solutions of* ($\mathrm{E}^{\prime}$) *are oscillatory*.

##### Remark 4

Similar comments to those in Remark 2 can be made for Theorems 6, 7 and 8, concerning equation ($\mathrm{E}^{\prime}$).

#### Difference inequalities

A slight modification in the proofs of Theorems 2-4 and 6-8 leads to the following results about deviating difference inequalities.

##### Theorem 9

*Assume that all conditions of Theorem* 2 [6] *or* 3 [7] *or* 4 [8] *hold*. *Then*

(i) *the retarded* [*advanced*] *difference inequality*
  $$ \Delta x(n)+p(n)x\bigl(\tau(n)\bigr)\leq0, \quad n\in \mathbb{N}_{0}\ \bigl[ \nabla x(n)-q(n)x\bigl(\sigma(n)\bigr)\geq0, n\in \mathbb{N} \bigr] , $$
  *has no eventually positive solutions*;
(ii) *the retarded* [*advanced*] *difference inequality*
  $$ \Delta x(n)+p(n)x\bigl(\tau(n)\bigr)\geq0,\quad n\in \mathbb{N}_{0}\ \bigl[ \nabla x(n)-q(n)x\bigl(\sigma(n)\bigr)\leq0, n\in \mathbb{N} \bigr] , $$
  *has no eventually negative solutions*.

### Discussion

In the present paper we are concerned with the oscillation of a linear delay or advanced difference equation with non-monotone argument. New sufficient conditions have been established for the oscillation of all solutions of (E) and ($\mathrm{E}^{\prime}$). These conditions include (2.12), (2.30), (2.31), (2.35), (2.37) and (2.38) of Theorems 2, 3, 4, 6, 7 and 8, respectively, and are based on an iterative method.

The main advantage of these conditions is that they improve all the oscillation conditions in the literature. Conditions (2.12) and (2.35) improve the non-iterative conditions that are listed in the introduction, namely conditions (1.7), (1.10) and (1.22), respectively. This conclusion becomes evident immediately by inspecting the left-hand side of (2.12), (2.35) and the left-hand side of (1.7), (1.10) and (1.22).

The improvement of (2.12) and (2.35) as to the other iterative conditions, namely (1.12) (for $r>1$), (1.15) (for $\ell >1$), (1.18) (for $\ell>1$) and (1.24) (for $r>1$), (1.27) (for $\ell>1$), is that they require far fewer iterations to establish oscillation, than the other conditions. This advantage can easily be verified computationally, by running the Matlab programs and comparing the number of iterations required by each condition to establish oscillation (see Section 3).

Similar observations and comments can be made for conditions (2.30) and (2.37). It is to be pointed out that conditions (2.31) and (2.38) are of a type different from all the known oscillation conditions. Nevertheless, in Example 2, it is shown that (2.38) implies oscillation, while other known ones fail.

## Examples and comments

In this section, examples illustrate cases when the results of the present paper imply oscillation while previously known results fail. The examples not only illustrate the significance of main results, but also serve to indicate the high degree of improvement, compared to the previous oscillation criteria in the literature. All the calculations were made in Matlab.

### Example 1

Taken and adapted from [7]

Consider the retarded difference equation

**Figure 1:**
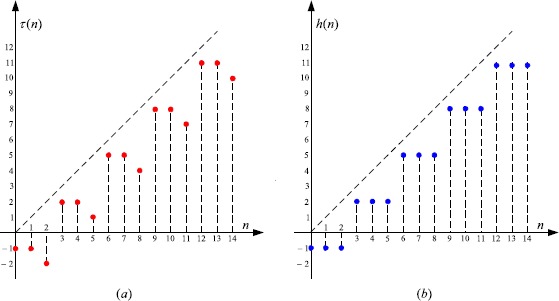
**The graphs of** $\pmb{\tau(n)}$ **and** $\pmb{h(n)}$ **.**

3.1 $$ \Delta x(n)+\frac{283}{2\text{,}000}x \bigl( \tau(n) \bigr) =0,\quad n \in \mathbb{N}_{0}, $$

with (see Figure 1(a))

$$ \tau(n)= \textstyle\begin{cases} n-1, & \text{if }n=3\mu, \\ n-2, & \text{if }n=3\mu+1, \\ n-4, & \text{if }n=3\mu+2, \end{cases}\displaystyle \quad\mu\in \mathbb{N}_{0}. $$

By (1.5), we see (Figure 1(b)) that

$$ h(n)=\max_{0\leq s\leq n}\tau(s)= \textstyle\begin{cases} n-1, & \text{if }n=3\mu, \\ n-2, & \text{if }n=3\mu+1, \\ n-3, & \text{if }n=3\mu+2, \end{cases}\displaystyle \quad\mu \in \mathbb{N} _{0}. $$

It is easy to see that

$$ \alpha=\liminf_{n\rightarrow\infty}\sum_{j=\tau (n)}^{n-1}p(j)= \liminf_{\mu\rightarrow\infty}\sum_{j=3\mu -1}^{3\mu-1} \frac{283}{2\text{,}000}=0.1415 $$

and, therefore, the smaller root of $e^{0.1415\lambda}=\lambda$ is $\lambda _{0}=1.18206$.

Clearly, $p(n)=\frac{283}{2\text{,}000}=0.1415<1/\lambda_{0}\simeq0.84598$, *i.e.*, (2.1) is satisfied.

Observe that the function $F_{\ell}:\mathbb{N} _{0}\rightarrow \mathbb{R} _{+}$ defined as

$$ F_{\ell}(n)=\sum_{i=h(n)}^{n}p(i)\exp \Biggl( \sum_{j=\tau (i)}^{h(n)-1}p(j)\prod _{m=\tau(j)}^{j-1}\frac{1}{1-\widetilde{P}_{\ell }(m)} \Biggr) $$

attains its maximum at $n=3\mu+2$, $\mu\in \mathbb{N} _{0}$, for every $\ell\in \mathbb{N} $. Specifically,

$$ F_{1}(3\mu+2)=\sum_{i=3\mu-1}^{3\mu+2}p(i) \exp \Biggl( \sum_{j=\tau(i)}^{3\mu-2}p(j)\prod _{m=\tau(j)}^{j-1}\frac {1}{1-\widetilde{P}_{1}(m)} \Biggr) $$

with

$$ \widetilde{P}_{1}(m)=p(m) \Biggl[ 1+\sum _{k=\tau(m)}^{m-1}p(k)\exp \Biggl( \sum _{w=\tau(k)}^{m-1}p(w)\prod_{v=\tau(w)}^{w-1} \frac {1}{1-\lambda_{0}p(v)} \Biggr) \Biggr] . $$

By using an algorithm on Matlab software, we obtain

$$ F_{1}(3\mu+2)\simeq1.0091 $$

and therefore

$$ \limsup_{n\rightarrow\infty}F_{1}(n)\simeq1.0091>1. $$

That is, condition (2.12) of Theorem 2 is satisfied for $\ell =1 $. Therefore, all solutions of equation (3.1) are oscillatory.

Observe, however, that

$$\begin{aligned}& \mathit{MD}=\limsup_{\mu\rightarrow\infty}\sum_{j=3\mu-1}^{3\mu +2}p(j)=4 \cdot\frac{283}{2\text{,}000}=0.566< 1, \\& \alpha=0.1415< \frac{1}{e}, \\& 0.566< 1-D(\alpha)\simeq0.9882, \\& \limsup_{n\rightarrow\infty }\sum_{j=h(n)}^{n}p(j) \prod_{i=\tau(j)}^{h(n)-1}\frac{1}{1-p(i)} \\& \quad = \limsup_{\mu\rightarrow\infty}\sum_{j=3\mu -1}^{3\mu+2} \frac{283}{2\text{,}000}\prod_{i=\tau(j)}^{3\mu-2} \frac {1}{1-\frac{283}{2\text{,}000}} \\& \quad = \frac{283}{2\text{,}000}\cdot\limsup_{\mu\rightarrow\infty } \Biggl\{ \prod _{i=\tau(3\mu-1)}^{3\mu-2}\frac{1}{1-\frac{283}{2\text{,}000}}+\prod _{i=\tau(3\mu)}^{3\mu-2}\frac{1}{1-\frac{283}{2\text{,}000}} \\& \qquad {}+\prod_{i=\tau(3\mu+1)}^{3\mu-2} \frac{1}{1-\frac{283}{2\text{,}000}}+\prod_{i=\tau(3\mu+2)}^{3\mu-2} \frac{1}{1-\frac{283}{2\text{,}000}} \Biggr\} \\& \quad = \frac{283}{2\text{,}000}\cdot\limsup_{\mu\rightarrow\infty } \Biggl\{ \prod _{i=3\mu-5}^{3\mu-2}\frac{1}{1-\frac{283}{2\text{,}000}}+\prod _{i=3\mu -1}^{3\mu-2}\frac{1}{1-\frac{283}{2\text{,}000}} \\& \qquad {}+\prod_{i=3\mu-1}^{3\mu-2} \frac{1}{1-\frac{283}{2\text{,}000}}+\prod_{i=3\mu -2}^{3\mu-2} \frac{1}{1-\frac{283}{2\text{,}000}} \Biggr\} \\& \quad = \frac{283}{2\text{,}000}\cdot\limsup_{\mu\rightarrow\infty } \biggl\{ \biggl( \frac{1}{1-\frac{283}{2\text{,}000}} \biggr) ^{4}+1+1+\frac{1}{1-\frac {283}{2\text{,}000}} \biggr\} \simeq0.7083< 1, \\& 0.7083< 1-D(\alpha)\simeq0.9882, \\& \limsup_{n\rightarrow\infty }\sum_{i=h(n)}^{n}p(i) \prod_{j=\tau(i)}^{h(n)-1}\frac {1}{1-p_{1}(j)} \simeq0.8169, \\& 0.8169< 1-D(\alpha)\simeq0.9882, \\& \limsup_{n\rightarrow\infty }\sum_{i=h(n)}^{n}p(i) \prod_{j=\tau(i)}^{h(n)-1}\frac {1}{1-P_{1}(j)} \simeq0.9550, \\& 0.9550< 1-D(\alpha)\simeq0.9882, \\& \limsup_{n\rightarrow\infty }\sum_{i=h(n)}^{n}p(i) \prod_{j=\tau(i)}^{n}\frac{1}{1-P_{1}(j)}\simeq 3.9361< \frac{1}{D(\alpha)}\simeq84.5735. \end{aligned}$$

That is, none of conditions (1.7), (1.8), (1.9), $\mbox{(1.10)} \equiv\mbox{(1.12)}$ (for $r=1$), $\mbox{(1.11)}\equiv \mbox{(1.13)}$ (for $r=1$), (1.15) (for $\ell=1$), (1.16) (for $\ell=1$), (1.18) (for $\ell=1$), (1.19) (for $\ell =1 $) and (1.20) (for $\ell=1$) is satisfied.

*Notation.* It is worth noting that the improvement of condition (2.12) to the corresponding condition (1.7) is significant, approximately 78.29%, if we compare the values on the left-side of these conditions. Also, the improvement compared to conditions (1.10), (1.15) and (1.18) is very satisfactory, around 42.47%, 23.53% and 5.67%, respectively.

Finally, observe that the conditions (1.12)-(1.13), (1.15)-(1.16) and (1.18)-(1.20) do not lead to oscillation for the first iteration. On the contrary, condition (2.12) is satisfied from the first iteration. This means that our condition is better and much faster than (1.12)-(1.13), (1.15)-(1.16) and (1.18)-(1.20).

### Example 2

Consider the advanced difference equation

**Figure 2:**
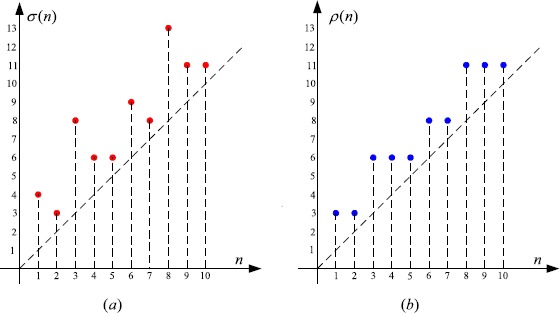
**The graphs of** $\pmb{\sigma(n)}$ **and** $\pmb{\rho(n)}$ **.**

3.2 $$ \nabla x(n)-\frac{297}{2\text{,}000}x \bigl( \sigma(n) \bigr) =0,\quad n\in \mathbb{N}, $$

with (see Figure 2(a))

$$ \sigma(n)= \textstyle\begin{cases} n+3, & \text{if }n=5\mu+1, \\ n+1, & \text{if }n=5\mu+2, \\ n+5, & \text{if }n=5\mu+3, \\ n+2, & \text{if }n=5\mu+4, \\ n+1, & \text{if }n=5\mu+5, \end{cases}\displaystyle \quad\mu\in \mathbb{N}_{0}. $$

By (1.6), we see (Figure 2(b)) that

$$ \rho(n)= \textstyle\begin{cases} n+2, & \text{if }n=5\mu+1, \\ n+1, & \text{if }n=5\mu+2, \\ n+3, & \text{if }n=5\mu+3, \\ n+2, & \text{if }n=5\mu+4, \\ n+1, & \text{if }n=5\mu+5, \end{cases}\displaystyle \quad\mu\in \mathbb{N}_{0}. $$

It is easy to see that

$$ \beta=\liminf_{n\rightarrow\infty}\sum_{j=n+1}^{\sigma (n)}q(j)= \liminf_{\mu\rightarrow\infty}\sum_{j=5\mu+3}^{5\mu +3}q(j)= \frac{297}{2\text{,}000}=0.1485. $$

Therefore, the smaller root of $e^{0.1485\lambda}=\lambda$ is $\lambda _{0}=1.194$ and

$$ \frac{1}{D ( \beta ) }-1\simeq75.0329. $$

Clearly, $q(n)=\frac{297}{2\text{,}000}=0.1485<1/\lambda_{0}\simeq0.8375$, *i.e.*, (2.33) is satisfied.

Observe that the function $F_{\ell}:\mathbb{N} _{0}\rightarrow \mathbb{R} _{+}$ defined as

$$ F_{\ell}(n)=\sum_{i=n}^{\rho(n)}q(i)\exp \Biggl( \sum_{j=n}^{\sigma(i)}q(j)\prod _{m=j+1}^{\sigma(j)}\frac {1}{1-\widetilde{Q}_{\ell}(m)} \Biggr) , $$

attains its maximum at $n=5\mu+3$, $\mu\in \mathbb{N} _{0}$, for every $\ell\in \mathbb{N} $. Specifically,

$$ F_{1}(5\mu+3)=\sum_{i=5\mu+3}^{5\mu+6}q(i) \exp \Biggl( \sum_{j=5\mu+3}^{\sigma(i)}q(j)\prod _{m=j+1}^{\sigma(j)}\frac {1}{1-\widetilde{Q}_{1}(m)} \Biggr) $$

with

$$ \widetilde{Q}_{1}(m)=q(m) \Biggl[ 1+\sum _{k=m+1}^{\sigma(m)}q(k)\exp \Biggl( \sum _{w=m+1}^{\sigma(k)}q(w)\prod_{v=w+1}^{\sigma (w)} \frac{1}{1-\lambda_{0}q(v)} \Biggr) \Biggr] . $$

By using an algorithm on Matlab software, we obtain

$$ F_{1}(5\mu+3)\simeq77.7076 $$

and therefore

$$ \limsup_{n\rightarrow\infty}F_{1}(n)\simeq77.7076> \frac{1}{D ( \beta ) }-1\simeq75.0329. $$

That is, condition (2.38) of Theorem 8 is satisfied for $\ell =1 $. Therefore, all solutions of equation (3.2) are oscillatory.

Observe, however, that

$$\begin{aligned}& \mathit{MA}=\limsup_{n\rightarrow\infty}\sum_{j=n}^{\rho (n)}q(j)= \limsup_{\mu\rightarrow\infty}\sum_{j=5\mu+3}^{5\mu +6}q(j)=0.594< 1, \\ & 0.594< 1- ( 1-\sqrt{1-\beta} ) ^{2}\simeq0.9940, \\ & \limsup_{n\rightarrow\infty}\sum_{j=n}^{\rho (n)}q(j)b_{1}^{-1} \bigl(\rho(n),\sigma(j)\bigr)\\ & \quad =\limsup_{\mu\rightarrow\infty }\sum _{j=5\mu+3}^{5\mu+6}q(j)b_{1}^{-1}\bigl(5 \mu+6,\sigma(j)\bigr) \\ & \quad = \frac{297}{2\text{,}000}\cdot\limsup_{\mu\rightarrow\infty}\bigl[ b_{1}^{-1}\bigl(5\mu+6,\sigma(5\mu+3)\bigr)+b_{1}^{-1} \bigl(5\mu+6,\sigma(5\mu +4)\bigr) \\ & \qquad {}+b_{1}^{-1}\bigl(5\mu+6,\sigma(5\mu+5) \bigr)+b_{1}^{-1}\bigl(5\mu+6,\sigma(5\mu +6)\bigr)\bigr] \\ & \quad = \frac{297}{2\text{,}000}\cdot\limsup_{\mu\rightarrow\infty}\bigl[ b_{1}^{-1}(5\mu+6,5\mu+8)+b_{1}^{-1}(5 \mu+6,5\mu+6) \\ & \qquad {}+b_{1}^{-1}(5\mu+6,5\mu+6)+b_{1}^{-1}(5 \mu+6,5\mu+9)\bigr] \\ & \quad = \frac{297}{2\text{,}000}\cdot \biggl[ \frac{1}{ ( 1-\frac{297}{2\text{,}000} ) ^{2}}+1+1+ \frac{1}{ ( 1-\frac{297}{2\text{,}000} ) ^{3}} \biggr] \simeq 0.7423< 1, \\ & 0.7423< 1-D(\beta)\simeq0.9868, \\ & \limsup_{n\rightarrow\infty}\sum_{i=n}^{\rho (n)}q(i) \prod_{j=\rho(n)+1}^{\sigma(i)}\frac{1}{1-q_{1}(j)}\simeq 0.9183< 1, \\ & 0.9183< 1-D(\beta)\simeq0.9868. \end{aligned}$$

That is, none of conditions (1.22), (1.23), (1.24) (for $r=1$), (1.25) (for $r=1$), (1.27) (for $\ell=1$) and (1.28) (for $\ell=1$) is satisfied.

*Notation.* It is worth noting that the conditions (1.24), (1.25), (1.27) and (1.28) do not lead to oscillation for the first iteration. On the contrary, condition (2.38) is satisfied from the first iteration. This means that our condition is better and much faster than (1.24), (1.25), (1.27) and (1.28).

### Remark 5

Similarly, one can construct examples, illustrating the other main results stated in the paper.

## Conclusions

In this paper, new sufficient oscillation conditions for all solutions of (E) and ($\mathrm{E}^{\prime}$) have been established. These conditions have been derived using an iterative technique. As a result, the conditions in this paper significantly improve on the previously reported conditions that are reviewed in the introduction. The results are illustrated by two examples, showing that our conditions achieve a significant improvement over the known conditions. That improvement gets even greater by appropriately selecting the coefficients $p(n)$ and $q(n)$ and the non-monotone arguments $\tau(n)$ and $\sigma(n)$.

The conditions in this paper involve limsup. Thus, an apparent research objective for future work can be establishing similar iterative techniques, for oscillation conditions, involving liminf.
